# A protocol to use *Drosophila melanogaster* larvae to model human glioblastoma

**DOI:** 10.1016/j.xpro.2022.101609

**Published:** 2022-08-08

**Authors:** Julia G. Saborio, Elizabeth E. Young, Alexander S. Chen, Renee D. Read

**Affiliations:** 1 Department of Pharmacology and Chemical Biology, Emory University, Atlanta, GA 30322, USA; 2 Graduate Program in Cancer Biology and Translational Oncology, Emory University, Atlanta, GA 30322, USA; 3 Department of Hematology and Medical Oncology, Emory University, Atlanta, GA 30322, USA; 4 Winship Cancer Institute, Emory University, Atlanta, GA 30322, USA

**Keywords:** Developmental biology, Cancer, Genetics, Model organisms

## Abstract

This protocol describes a genetic model system we developed for glioblastoma (GBM) in *Drosophila melanogaster*, which can be used to explore the pathogenic phenotypic effects of mutated genetic pathways and to identify potential therapeutic targets for tumors with these mutations. We present genetic schemes and experimental steps needed to create neoplastic glial brain tumors in larval *Drosophila*. We also provide steps to manipulate genes in this model and to perform brain fixation, immunostaining, and imaging of neoplastic larval brains.

For complete details on the use and execution of this protocol, please refer to Read et al., (2009).

## Highlights

• Create genetic models of glioblastoma in *Drosophila melanogaster* using UAS-Gal4 systems
• Test RNAi, overexpression, or mutant variants of genes of interest in neoplastic glia
• Immunostain and image neoplastic brains, analyze data qualitatively and quantitatively
• Explore effects of genetic modifiers and mutations in intact *Drosophila* nervous system

## Abstract (editor highlights)

Publisher’s note: Undertaking any experimental protocol requires adherence to local institutional guidelines for laboratory safety and ethics.

## Graphical abstract

**Figure:**
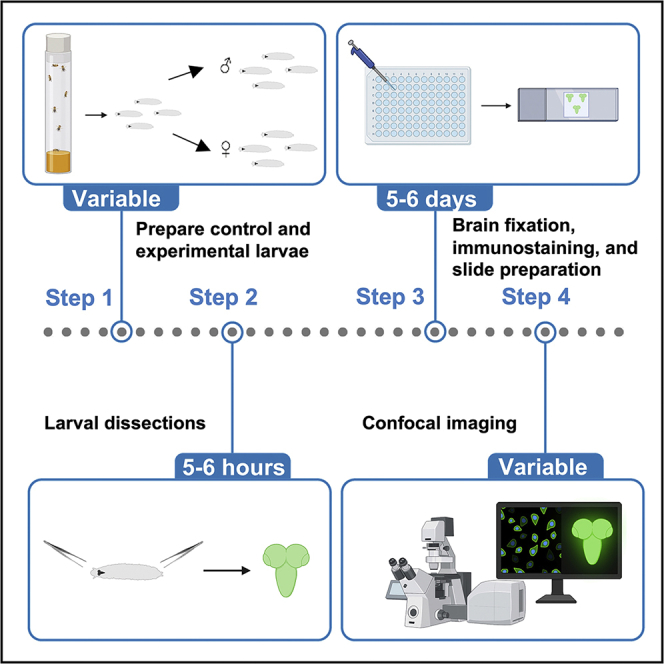

## Before you begin

The protocol below describes how to use *Drosophila melanogaster* as an effective model for glioblastoma (GBM) tumor cell biology and genetics (Chen and Read, 2019). Humans and *Drosophila* have extensive cellular and genetic homology, human glial cells have *Drosophila* counterparts, and many human genes of interest in GBMs have functional *Drosophila* orthologs. Mutations that cause amplification, constitutive activation, and overexpression of receptor tyrosine kinases (RTKs) and other genes in RTK signaling pathways are frequently altered in GBM tumors (Brennan et al., 2013). In *Drosophila*, activation of RTKs and downstream Phosphoinositide 3-kinase (PI3K) signaling pathways leads to development of glial tumors with key features of human gliomas and can therefore be used to study how genetic manipulation affects proliferation and differentiation of neoplastic glial cells (Read et al., 2009). Our GBM model allows for cell-type specific tissue manipulation by expression of constitutively active forms of the RTK epidermal growth factor receptor (EGFR) and dp110, a subunit of PI3K, both of which are commonly mutated in human GBM tumors to drive tumorigenesis. Together, these two mutations recreate the GBM phenotype in the fly larval brain and cause late larval lethality. In comparison with mouse models, *Drosophila* are relatively inexpensive, have a short life span, and are easily manipulated with powerful genetic tools. In addition, by applying forward genetic screens of mutations or overexpression constructs, this model can be expanded to discover novel genes that drive glial neoplasia and to elucidate other downstream players in the EGFR and PI3K pathways that act as “enhancers,” which produce a worse GBM phenotype, or “suppressors,” which ameliorate the GBM phenotype (Read et al., 2013). This protocol will cover how to use this model to assess the effects of genetic interventions on the GBM phenotype in the larval *Drosophila* brain.

### Institutional permissions

Any experiments on live *Drosophila melanogaster* must be performed in accordance with relevant institutional and national guidelines and regulations. The experiments described below were reviewed and approved by the Emory University Institutional Biosafety Committee on Research Safety (protocol #B6-272-12R21).

### Generating stable *Drosophila* stocks for the glioblastoma model

**Timing: 3–10 weeks**

**Figure 1:**
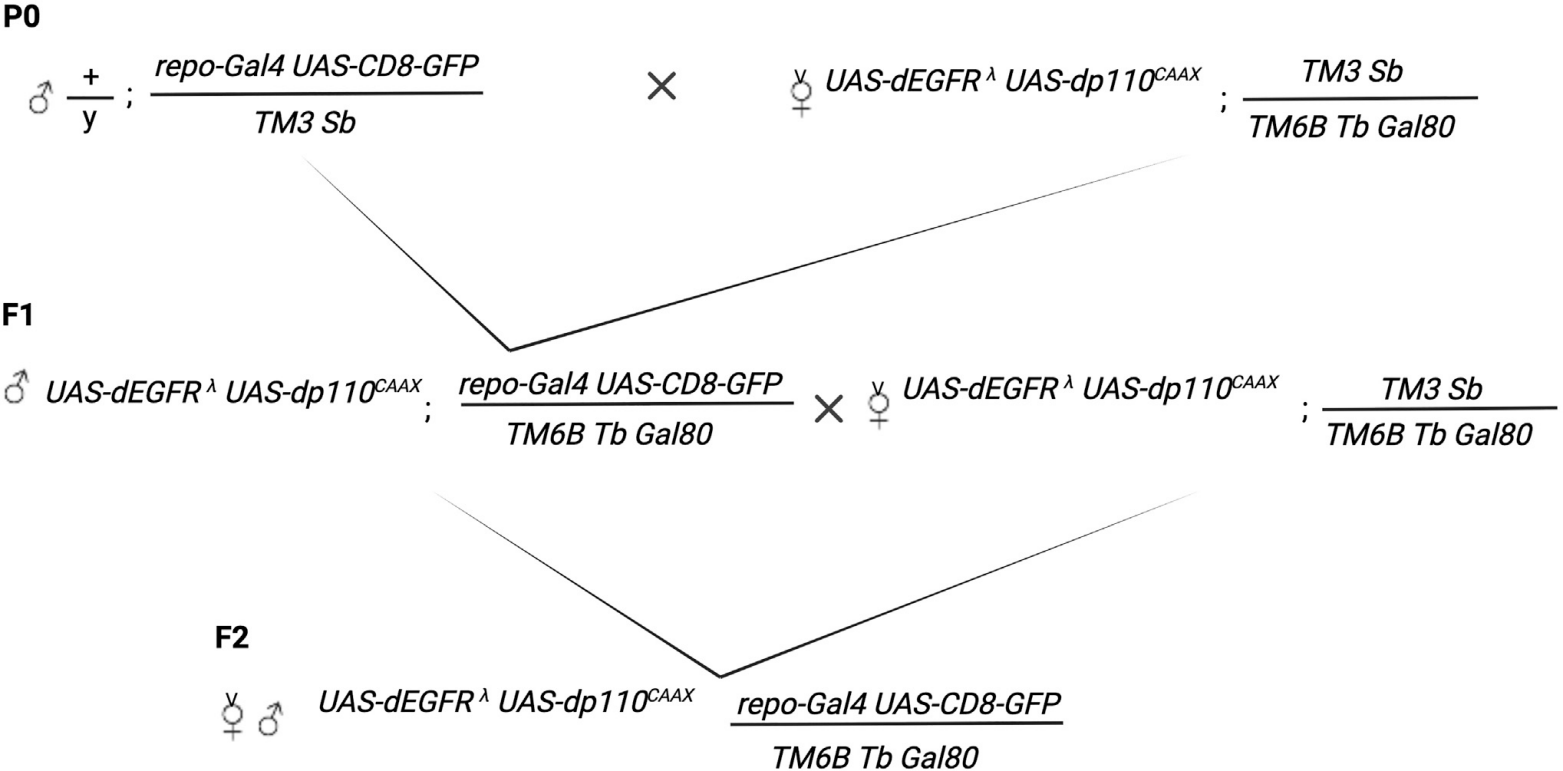
Genetic crossing scheme to create a *Drosophila* EGFR-PI3K GBM model Genetic crossing scheme used to derive the stable experimental stock described in the protocol. *TM3 Sb* and *TM6B Tb Gal80* are balancer chromosomes and markers used to select progeny of the desired genotype. *repo-Gal4* is expressed in a glial specific manner throughout larval life. *UAS-dEGFR*^*λ*^*UAS-dp110*^*CAAX*^ are transgene constructs are expressed by *repo-Gal4* to drive GBM-like glial neoplasia. *UAS-CD8-GFP* provides a glial specific GFP label for neoplastic glia.

Here, we describe a multi-step crossing scheme to generate a stable stock homozygous for *UAS*-*EGFR*^*λ*^ and *UAS-dp110*^*CAAX*^ oncogenic transgenes recombined on the X chromosome, with *repo-Gal4* and *UAS-CD8-GFP* to drive oncogene expression and mark glial cells with membrane-bound GFP maintained over a TM6B Tb balancer with tub-Gal80 (Read et al., 2013) (Figure 1). This stock will then be used as the P0 parental stock for crosses to create experimental F1 larvae with neoplastic brains.

***Note:*** All flies are grown on a standard cornmeal medium (recipe included).

1. Recombine *UAS-dEGFR*^*λ*^ and *UAS-dp110*^*CAAX*^ oncogene transgenes onto the X chromosome.
2. Recombine *repo-Gal4* and *UAS-CD8-GFP* onto the 3^rd^ chromosome.
3. Create *UAS-dEGFR*^*λ*^ *UAS-dp110*^*CAAX*^; *TM3 Sb/ TM6B Tb tub-Gal80* by standard crossing; be mindful that this will require backcrossing given that the *UAS-dEGFR*^*λ*^ *UAS-dp110*^*CAAX*^ transgenes are on the X chromosome.
4. Create *UAS-dEGFR*^*λ*^ *UAS-dp110*^*CAAX*^; *repo-Gal4 UAS-CD8-GFP/ TM6B Tb tub-Gal80* by standard crossing between *UAS-dEGFR*^*λ*^ *UAS-dp110*^*CAAX*^ ; *TM3 Sb/ TM6B Tb tub-Gal80* and *repo-Gal4 UAS-CD8-GFP/TM3 Sb* stocks.

***Note:****UAS-dEGFR*^*λ*^*UAS-dp110*^*CAAX*^; *repo-Gal4 UAS-CD8-GFP/TM3 Sb* is larval/pupal lethal and the *UAS-dEGFR*^*λ*^*UAS-dp110*^*CAAX*^; *repo-Gal4 UAS-CD8-GFP/ TM6B Tb tub-Gal80* is viable such that only *TM6B Tb tub-Gal80* balanced animals will survive to adulthood, which allows for quick stock generation. This is because Gal80 expressed from the *TM6B Tb tub-Gal80* balancer suppresses overexpression of the *UAS-dEGFR*^*λ*^ and *UAS-dp110*^*CAAX*^ transgenes. Confirmation of *TM6B Tb tub-Gal80* is done using the Tb body morphology marker.

### Preparing experimental flies

**Timing: 2–3 weeks**

These steps outline a one-step cross to test the phenotypic impact of an additional transgene or a mutation of interest in 3^rd^ instar larvae in which the *dEGFR*^*λ*^ and *dp110*^*CAAX*^ oncogenes are overexpressed under the control of the glia-specific driver *repo-Gal4*.

5. Collect virgins from the *UAS-dEGFR*^*λ*^ *UAS-dp110*^*CAAX*^; *repo-Gal4 UAS-CD8-GFP*/ *TM6B Tb tub-Gal80* stock.

***Note:*** In crosses, the *Tmb6B Tb tub-Gal80* balancer segregates away from *repo-Gal4*, leading to glial-specific expression of CD8-GFP, dEGFR^λ^ and dp110^CAAX^ oncogenes, and experimental transgenes (e.g., genetically encoded RNAi constructs) in F1 progeny.

6. Set up all experimental tester crosses and control crosses as follows:
  a. Use a minimum of 20–25 young *UAS-dEGFR*^*λ*^ *UAS-dp110*^*CAAX*^; *repo-Gal4 UAS-CD8-GFP/ TM6B Tb tub-Gal80* virgin females with 10–25 males per vial and incubate crosses at 25°C.
  b. After 72 h of initial incubation to allow for mating, to create age-matched F1 larval progeny, flip the parental adults into fresh vials every 24 h and maintain the F1 cross progeny at 25°C for 5–6 days (120–144 h) (Read et al., 2013).
  c. Collect and dissect age-matched 120–144-h old wandering 3^rd^ instar larvae.
7. Set up vials of each experimental tester crosses with virgin females of the stable oncogene stock (genotype: *UAS-dEGFR*^*λ*^ *UAS-dp110*^*CAAX*^*; repo-Gal4 UAS-CD8-GFP/ TM6B Tb tub-Gal80*) and males with the desired transgene or mutation.

***Note:*** For parental males with autosomal homozygous mutations or transgenes, all non-Tb F1 larvae will contain the desired mutation in the presence of the *UAS-dEGFR*^*λ*^*UAS-dp110*^*CAAX*^*; repo-Gal4 UAS-CD8-GFP* constructs. Homozygous lethal mutations or transgenes should be balanced in parental males using markers that are clearly identifiable in larvae to allow for unambiguous genotyping of F1 larval progeny.

**Figure 2:**
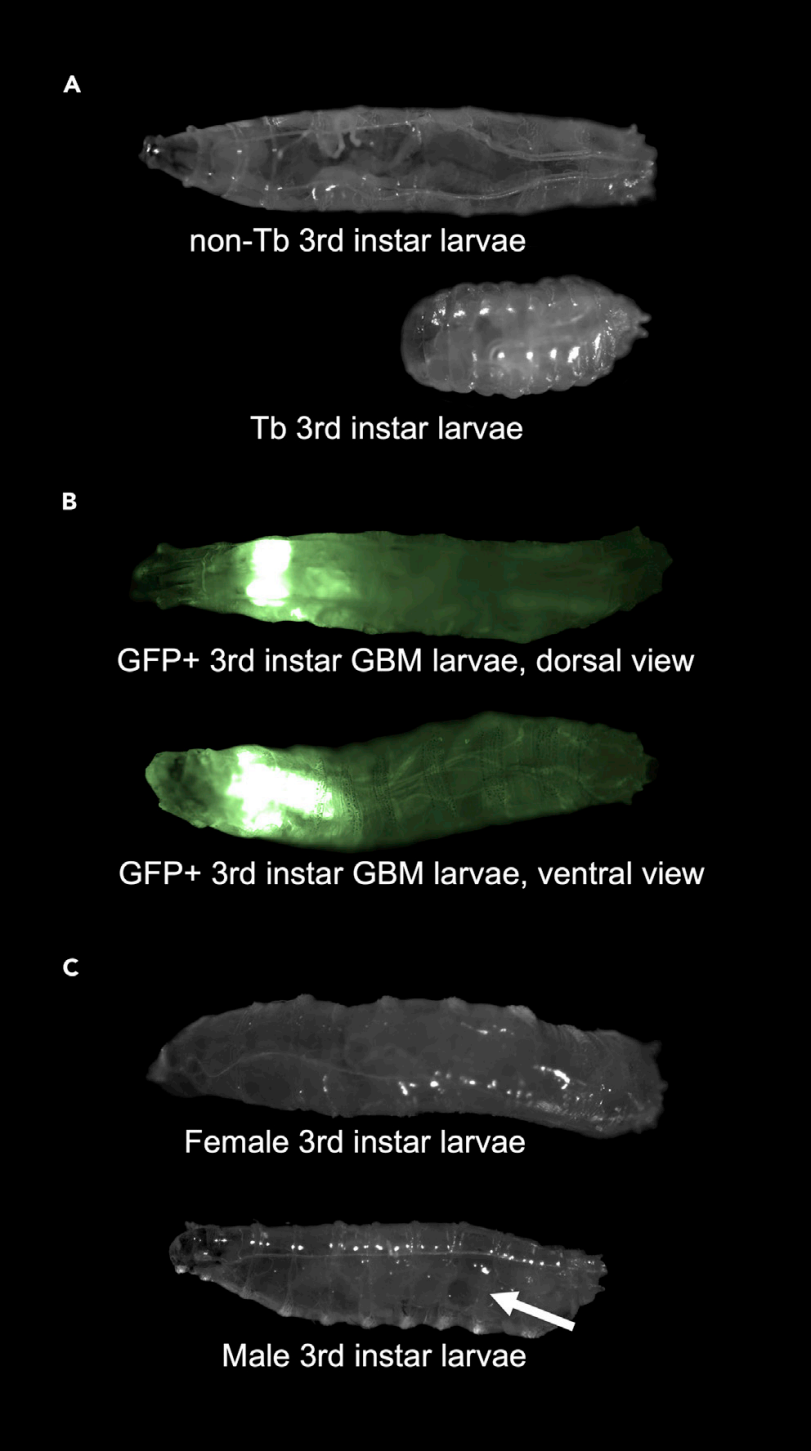
Markers to genotype larvae in the EGFR-PI3K GBM model (A) The *repo>dEGFR*^*λ*^*; dp110*^*CAAX*^ model is maintained over a *TM6BTb tub-Gal80* balancer chromosome, which yields two classes of F1 progeny once the stable P0 stock is outcrossed to control or experimental tester genotypes: one with the desired genotype in which repo-Gal4 is activated to drive *dEGFR^λ^;dp110*^*CAAX*^ overexpression (upper), the other with the *TM6BTb* balancer (lower). (B) 3^rd^ instar wandering larvae showing *repo>CD8-GFP* epifluorescence in neoplastic *dEGFR*^*λ*^*;dp110*^*CAAX*^ glial cells, as visualized in live animals, in two different views. (C) Male and female 3^rd^ instar larvae can be distinguished by the presence of male gonads, emphasized by the arrow. Gonads will be visible as translucent circles on the posterior half of male larvae.

***Note:*** The inclusion of a *UAS-CD8-GFP* transgene in the stable parental stock will result in *repo-Gal4*-driven expression of membrane-bound GFP in all *dEGFR*^*λ*^ and *dp110*^*CAAX*^ overexpressing glial cells in F1 progeny, which enables easy identification of affected larvae using GFP epifluorescence and facilitates their phenotypic analyses (Figure 2B).

8. In parallel with the experimental tester crosses, set up positive control GBM model larvae, which should be created from virgin females of the stable oncogene stock (genotype: *UAS-dEGFR*^*λ*^ *UAS-dp110*^*CAAX*^*; repo-Gal4 UAS-CD8-GFP/ TM6B Tb tub-Gal80,* Figure 1) and wild-type or control strain males.
  a. Use males homozygous for a *UAS-LacZ* transgene to control for the number of UAS transgenes in the GBM model.
  b. To create control age-matched control larvae for various mutant alleles, use *w*^*1118*^ males or males from other matched genetic backgrounds can be mated to virgin females of the stable oncogene stock.
9. Grow F1 cross progeny in vials for 120–144 h (5–6 days) and select only the non-Tb 3^rd^ instar wandering larvae for dissection (Figure 2A).

***Optional:*** To separate larvae by sex, look for the presence of male gonads, which resemble large translucent circles on both sides of male larvae, closer to their posterior end (Figure 2C) (Karabasheva and Smyth, 2020). Females generally have much smaller gonads that are not as easily visible as male gonads. In addition, with our fly food recipe and culture conditions, male larvae are usually smaller than their female counterparts, and male larvae often begin wandering later than male larvae (Karabasheva and Smyth, 2020).

**CRITICAL:** For all crosses, the GBM phenotype is dynamic, and related to the growth rate of neoplastic glia under specific culture conditions such that experimental phenotypes caused by an introduced transgene or mutation, such as a UAS-dsRNA construct, should be compared to phenotypes of age-matched control animals with appropriate control UAS transgenes or mutations. To ensure age-matching between larvae and to avoid overcrowding of larvae, flip the parent cross into a fresh food every 12–24 h. Conversely, to ensure adequate numbers of F1 larvae, add yeast to cross vials to stimulate egg laying by parental females and, as the parental animals age, add fresh virgin females or males to crosses in order to maintain the appropriate number of parental animals to generate fresh F1 larval progeny for dissection.

## Key resources table

**Table:** 

REAGENT or RESOURCE	SOURCE	IDENTIFIER
**Antibodies**		

Mouse anti-Repo monoclonal, primary antibody, to label glial cell nuclei, final dilution 1:10	Developmental Studies Hybridoma Bank	Cat #8D12
Alexa Fluor® 594 AffiniPure F(ab')₂ Fragment Donkey Anti-Mouse IgG (H+L), final dilution 1:400	Jackson Immunolabs	Cat #715-586-151
Alexa Fluor® 647 AffiniPure Goat Anti-Horseradish Peroxidase, to label neuronal cell types, final dilution 1:100	Jackson Immunolabs	Cat #123-605-021

**Chemicals, peptides, and recombinant proteins**		

10× Phosphate-buffered saline (PBS) pH 7.4	Cellgro	Cat #46-013-CM
32% paraformaldehyde	Electron Microscopy Sciences	Cat #15714
Triton X-100	Santa Cruz Biotechnology	Cat #sc-29112
1% sodium azide	G-Biosciences	Cat #786-299
Normal goat serum	Cell Signaling Technology	Cat #5425
Vectashield® Antifade Mounting Medium	Vector Laboratories	Cat #H-1000
Vectashield® Antifade Mounting Medium with DAPI	Vector Laboratories	Cat #H-1200

**Experimental models: Organisms/strains**		

*UAS-dEGFR*^*λ*^ on X chromosome *Drosophila* stock (Read et al., 2009)	Dr. Gertrud Schupbach (schupbac@princeton.edu)	On request
*UAS-dp110*^*CAAX*^ on X chromosome *Drosophila* stock (Read et al., 2009)	Bloomington Drosophila Stock Center	Stock# 8294
*UAS-CD8-GFP* on 3^rd^ chromosome	Bloomington Drosophila Stock Center	Stock# 5130
*UAS-lacZ* on 2^nd^ chromosome	Bloomington Drosophila Stock Center	Stock# 3955
*repo-Gal4* on 3^rd^ chromosome	Bloomington Drosophila Stock Center	Stock# 7415
*TM6B Tb Tub-Gal80*	Bloomington Drosophila Stock Center	Stock# 9490

**Software and algorithms**		

Photoshop	Adobe	https://www.adobe.com/
Imaris	Oxford Instruments	https://imaris.oxinst.com/newrelease

**Other**		

Pyrex glass 9-well spot test plate	Corning	Cat# 7720-85
Bamboo Splints 6″ long	Ted Pella, Inc.	Cat #116
Dumont Inox Forceps #5	Fine Science Tools	Cat# 11252-20
Dumont Inox Forceps #55	Fine Science Tools	Cat# 11255-20
no. 11 Feather surgical blade	Graham-Field	Cat# 2976#11
96-well assay plate (no lid)	Corning	Cat# 2595
Scotch transparent tape	3 M	Cat# 600
Coverslips, No. 1 thickness	VWR	Cat# 48366067
Glass microscope slides	Fisherbrand	Cat# 12-550-15
Clear nail polish	Sally Hansen	N/A
Laser Scanning Confocal microscope	Varies: ZEISS, Olympus, Nikon are examples	LSM 700, LSM 900, FluoView 1200, or equivalent system
Molasses, 5 Gallons (18.93 L), Fly Food Ingredient	Genesee Scientific	Cat #62-118
Drosophila Agar (25 g)	Genesee Scientific	Cat #66-105
Tegosept, 5 Kg, Fly Food Preservative (mold inhibitor)	Apex Bioresearch Products	Cat #20-259
Yellow Cornmeal (11.3 Kg), Medium Grind	Genesee Scientific	Cat #62-101
Inactive Dry Yeast, Nutritional Flake	Genesee Scientific	Cat #62-108
Propionic Acid 99% 2500 mL - AC14930-0025, 2.5 L	Acros Organics	Cat #AC14930-0025
Drosophila Vials, Wide (PS), Polystyrene, Tray	Genesee Scientific	Cat #32-110
Flugs® - Wide Plastic Vials, Drosophila Closures [AS275]	Genesee Scientific	Cat #49-101

## Materials and equipment

**Table:** *Drosophila* Cornmeal Medium

Reagent	Final concentration	Amount
Cornmeal	–	70 g
Yeast	–	28 g
Agar	–	14 g
Molasses	–	87.5 mL
tegosept (mold inhibitor)	–	2.3 g
Ethanol	–	23 mL
propionic acid (13.4 M, 99.9%)	0.042 M	5 mL
Water	–	to volume
**Total**	**N/A**	**1.6 L**

Store ingredients at 20°C. Prepare growth medium in kettle. Add to kettle while stirring on high: cold water (1 L), cornmeal, yeast, agar, and molasses. Turn on the steam and stirrer and get food to boil. Simmer 30 min while stirring on high until food is thick and smooth. Stir while adding to kettle: mold inhibitor (10% w/v in 23 mL ethanol) and propionic acid. Stir and add water to total 1.6 L final volume. Cook, stirring vigorously, for 10 min. Turn off heat. Pour food into vials or bottled using a filler, cool food and allow agar to set, plug vials and bottles with flugs or cotton. The maximum storage time for individual reagents is provided by vendors. The maximum storage time for fly food is approximately 3 months when stored at 4°C.

**CRITICAL:** Propionic acid is hazardous and can irritate and burn skin and damage eyes and may cause respiratory irritation. Wash skin and eyes thoroughly after exposure. Both propionic acid and ethanol are flammable as liquid and vapor; store in flammable storage cabinet under fume hood. Do not use in the presence of open flame. Ethanol can be toxic; do not ingest.

**Table:** 1× PBS + 0.3% Triton X-100 (PBS .3% Triton)

Reagent	Final concentration	Amount
10× Phosphate Buffered Saline (PBS), pH 7.4	1×	5 mL
Triton X-100	0.3%	150 μL
MilliQ H_2_O	N/A	45 mL
**Total**	**N/A**	**50 mL**

Store at 20°C.

**Table:** Primary antibody staining solution

Reagent	Final concentration	Amount
1× PBS pH 7.4, .3% Triton	1×	750 μL
Normal goat serum (NGS)	10%	100 μL
Anti-repo (glial cell nuclei)	1:10	100 μL
1% sodium azide	.05%	50 μL
**Total**	**N/A**	**1 mL**

Primary antibody staining solution can be reused approximately 4 times. Store antibodies and NGS at 4°C or −20°C according to manufacturer’s instructions.

**CRITICAL:** Sodium azide is a hazardous chemical that can be toxic through skin contact, inhalation, and ingestion. Do not inhale or ingest, thoroughly rinse following skin or eye contact. Using a premade 1% solution minimizes exposure and contamination risks.

**Table:** Secondary antibody staining solution

Reagent	Final concentration	Amount
1× PBS pH 7.4, .3% Triton	1×	827.5 μL
Normal goat serum (NGS)	10%	100 μL
anti-HRP-647	1:50	20 μL
anti-mouse-Alexa-594	1:400	2.5 μL
1% sodium azide	.05%	50 μL
**Total**	**N/A**	**1 mL**

Solution should not be reused but excess can be stored at 4°C protected from light and used within 5 days. Store antibodies and NGS at 4°C or −20°C according to manufacturer’s instructions.

**CRITICAL:** Sodium azide is a hazardous chemical that can be toxic through skin contact, inhalation, and ingestion. Do not inhale or ingest, thoroughly rinse following skin or eye contact. Using a premade 1% solution minimizes exposure and contamination risks.

## Step-by-step method details

### *Drosophila melanogaster* 3^rd^ instar larvae whole brain dissection

**Timing: 3 days**

We first dissect whole brains from 3^rd^ instar larvae. This step is repeated on 3 consecutive days, and the vials that larvae are collected from must be age-matched at 5–6 days old.

**Figure 3:**
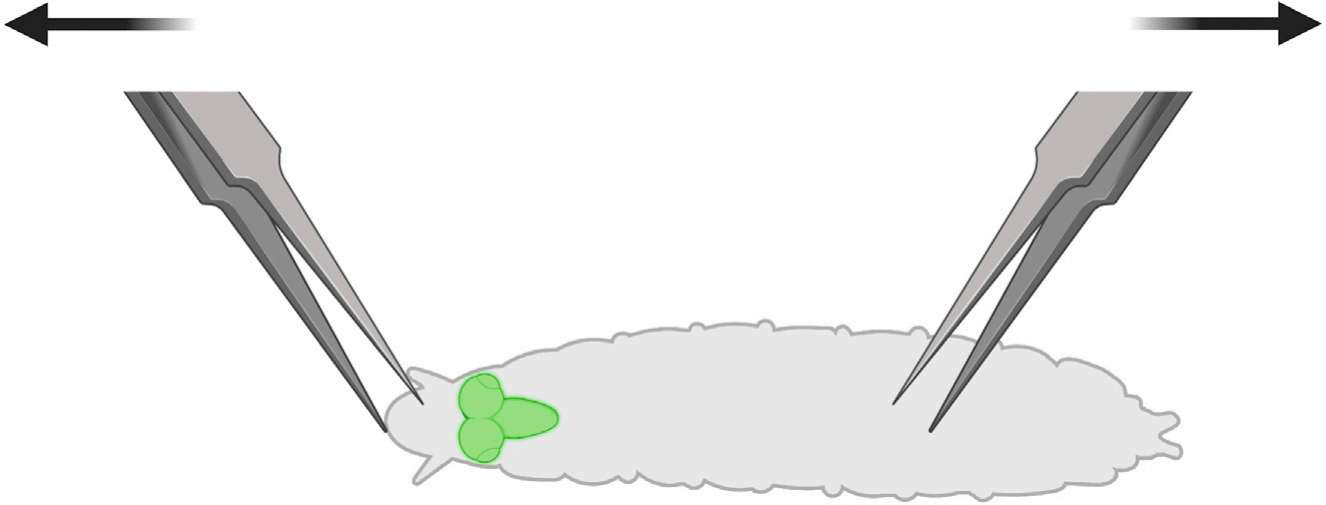
Larval brain dissection for the EGFR-PI3K GBM model 3^rd^ instar wandering larvae show neoplastic *dEGFR^λ^;dp110*^*CAAX*^ brains labeled by *repo>CD8-GFP* epifluorescence. For dissections of these larvae, pinch mouth parts with one pair of Inox forceps and pinch the body with another pair of Inox forceps, and pull apart to break open the body and release the nervous system.
5. Gently pull larvae apart to allow CNS and surrounding tissue to spill out into the PBS pool.
6. Identify the larval brain, which is attached to the mouth parts, and remove excess larval tissues while maintaining a grip on the mouth parts, transferring samples using mouth parts.

***Note:*** The gut and pharynx remnants should be removed to avoid possible contamination from bacteria, yeast, and food from within the digestive track.

1. Prepare one 9-well spot plate per phenotype with 750 μL 1× PBS in each spot.
2. From age-matched experimental tester and control vials, select wandering 3^rd^ instar F1 larvae at the desired time point and place into the top left well.
3. Rinse the larvae in 1× PBS and separate by sex into the following rows.
  a. Swirl the larvae in 1× PBS in the well, then transfer to the next well and swirl again to remove excess food and other contaminants from the animal bodies.
  b. Under a dissecting stereomicroscope, separate the larvae by sex if desired through identification of gonads on male larvae. Place male and female larvae into the first well of their corresponding row. Refer to Figure 2C for details on sex identification.
4. To begin dissecting, transfer larvae into a clean well and submerge with fresh 1× PBS. Using two pairs of Inox forceps under the microscope, grasp the larvae’s mouth parts using one hand and the lower third of the larval body with the other pair of forceps using the other hand (Figure 3).

7. When the brain has been dissected, transfer it into a fresh well with 1× PBS. Repeat the dissection with the rest of the larvae.
8. Once the desired number of larval brains are collected, prepare a 96-well plate with separate wells for each genotype, and separate by males and females if desired (Figure 4). Pipette 100 μL of freshly prepared 1× PBS 4% paraformaldehyde into each well that will be used, then transfer the brains into the corresponding well.

**Figure 4:**
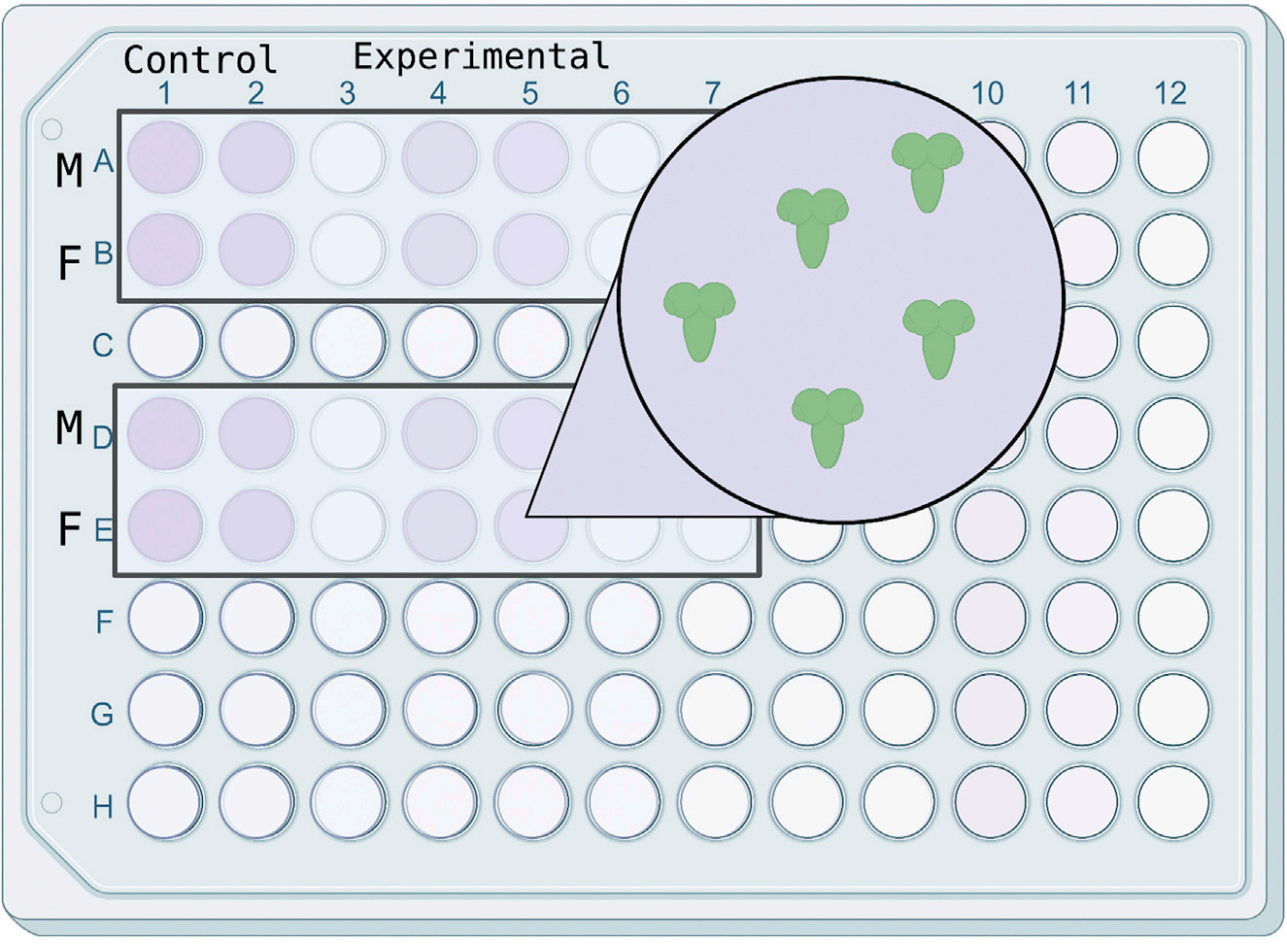
Larval brain immunostaining 96-well plate set up for larval brain immunostaining and storage. Wells containing tissue are denoted in purple. Tape to create an airtight seal as shown.
9. Allow the samples to fix for 30–60 min.
10. After fixation, aspirate the 1× PBS 4% paraformaldehyde using a pipette, being careful not to aspirate or damage the tissue. Discard 4% paraformaldehyde with hazardous chemical waste.
11. Wash the tissue with 100 μL 1× PBS 0.3% Triton immediately after removing the fixative, and incubate the samples for 10 min. Repeat this step 2 times for a total wash time of 30 min.
12. Following the final wash, replace with 100 μL 1× PBS 0.3% Triton and 1 pipette drop of 1% sodium azide solution (5–10 μL) to a final concentration of 0.05%–0.1% for preservation.
13. Using clear tape, tape over the wells in use to create an air-tight seal. Place the plate into a 4°C storage and leave for 24 h.

**Pause point:** Preserved fixed brains may be stored at 4°C long-term (up to 1 year or more) in this way and reserved for future staining.

### Immunofluorescence staining of *Drosophila melanogaster* whole larval brains

**Timing: 4 days**

In this section, we outline the steps for immunohistochemical staining of *Drosophila* brains using mammalian antibodies. Below, we describe stain procedures for anti-Repo labeling in order to detect all glial cell nuclei. Other primary antibodies or immunofluorescence stains may be used to detect other proteins or genes of interest.

14. Remove the dissected larval brains from 4°C storage and remove tape.
15. Under a stereomicroscope, aspirate the 1× PBS 0.3% Triton sodium azide solution from each well to be stained.
16. Replace with 100 μL 1× PBS 0.3% Triton 10% NGS 0.1% sodium azide to pre-block the tissue prior to primary antibody staining. Incubate in blocking solution for 30 min.
17. After blocking, add 50–100 μL primary antibody staining solution. Ensure all tissue is completely submerged in the stain and transparent tape over the plate wells to create an airtight seal.

***Optional:*** To visualize changes in glial cell numbers, use the anti-Repo primary antibody (1:10, Developmental Studies Hybridoma Bank) which specifically detects a glial-specific transcription factor expressed in *repo-Gal4* positive glia and labels neoplastic all glial cell nuclei (Read et al., 2009).

18. Incubate brains in primary antibody in 4°C for 48 h.

***Note:*** We have found that longer incubation times of 48–72 h allow primary antibodies to equilibrate deeper into neoplastic brains, which can be denser and thicker than normal wild-type 3^rd^ instar larval brains. Incubation times of 24 h or less does not allow sufficient time for primary antibodies to fully stain neoplastic glia located deeper within tumorous larval brains.

19. Remove brains from the 4°C, remove the tape, and aspirate the primary antibody stain.

***Note:*** Primary antibody solution may be collected, stored at 4°C, and reused to stain other samples as warranted.

20. Wash the tissue after staining in primary antibodies with 100 μL 1× PBS 0.3% Triton wash solution and incubate for 10 min. Repeat the wash step 3 times, for a total wash time of 30 min.
21. Replace the final 1× PBS 0.3% Triton wash with 50–100 μL secondary antibody staining solution. Ensure all tissue is completely submerged in the staining solution.

***Note:*** Use F(ab’)_2_ fragments as secondary antibodies to ensure optimal penetration of staining into neoplastic tissues. Recommended secondary F(ab’)_2_ antibodies are shown in the antibody table above.

***Optional:*** To counterstain neuronal cell types during the secondary antibody staining step, use a fluorescently conjugated anti-HRP secondary antibody to mark neuronal cell bodies and neuronal fibers (neuropil) (Read et al., 2009).

22. Tape over the wells to create an airtight seal and store at 4°C for 24 h.
23. Remove the tape and aspirate the secondary antibody stain.
24. Wash the tissue after staining in secondary antibodies with 100 μL 1× PBS 0.3% Triton wash solution and incubate for 10 min. Repeat the wash step 3 times, for a total time of 30 min wash time.
25. Aspirate final wash solution resuspend the brains in Vectashield antifade and mounting media.
26. Cover the wells with transparent tape and store at 4°C to allow brains to clear for 24 h.

***Note:*** We prefer to use Vectashield antifade and mounting media with DAPI, which will cause nuclei to fluoresce in the UV channel, and can be used to image all cell nuclei. DAPI may have spectral overlap with other fluorescent stains, which should be factored into planning all staining and imaging procedures. If DAPI is not required or will overlap with other immunofluorescence stains, we recommend Vectashield antifade mounting media without DAPI.

**Pause point:** Whole larval brains may be stored in Vectashield at 4°C for up to 3–6 months for future imaging at this stage, depending on the stability of the primary antibody staining.

### Imaging *Drosophila melanogaster* brains using a confocal microscope

**Timing: 1 h**

These steps outline the optimal way to mount slides and prepare them for imaging.

**Figure 5:**
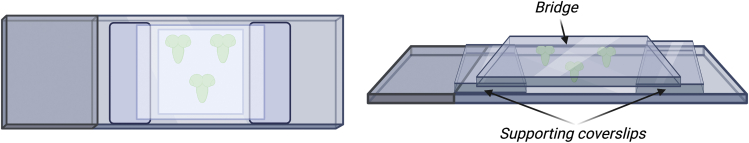
Mounting larval brains on slides for confocal imaging Slide mount set up to create a Vectashield chamber to preserve and orient brain tissue for imaging. A coverslip is snapped in half to create the supporting coverslips, and a bridge is created by putting another coverslip over these and sealing with nail polish. To ensure proper seating of the coverslips and firm sealing of the bridge, do not overfill the chamber with Vectashield.

***Note:*** If the Vectashield does not fill the chamber under the bridged coverslip, more Vectashield can be added with a micropipette, but do not completely fill or overfill the chamber since that will interfere with proper sealing.

27. Prepare a microscope slide with approximately 100 μL of Vectashield. Several brains can be mounted on one slide.
28. Remove immunofluorescence-stained brains from storage and remove tape. Using forceps or a pipette tip, transfer the brains to the microscope slide in Vectashield.
29. Under a dissecting stereomicroscope, hold associated excess larval tissue with forceps and use a surgical razor blade to carefully cut away all excess tissues (imaginal discs, salivary glands) from the brain, including the mouth parts.
30. To keep the imaging field clean, use forceps to remove any excess tissue residue from the slide.
31. When all brains have been prepared and cleaned, place them all dorsal side up towards the coverslip/ventral side down on the slide, and center brains on the slide.
32. To support a bridge coverslip that will make a chamber that will protect the brain tissues from being crushed, break a coverslip in half and place each half on each side of the Vectashield pool on the microscope slide, with the flat edges facing the brains.
33. Carefully lay another coverslip on top of the two halves to create a bridge that will create a chamber that will preserve brain morphology for imaging (Figure 5). Let the coverslips sit for at least 10 min before sealing as described below so that the Vectashield spreads on the glass slide and equilibrates with the coverslips.

34. Using clear nail polish, seal the edges of the bridge around the Vectashield chamber and seal the coverslips to the slide. Let dry.

***Note:*** Check that coverslips are secure prior to imaging and/or storing. If coverslips are not secure, they can drift during imaging which will shear and damage brain samples. To adjust coverslips, use a razor blade to remove nail polish, blot excess Vectashield, adjust coverslips with forceps, and re-seal.

35. Slides can be stored flat at 4°C in a slide holder.

**Pause point:** Slides may be stored at 4°C for up to 2 months or more, depending on whether/how long alcohols from the nail polish leach into the Vectashield and distorts staining of the tissues.

36. Image and optically section brains using a laser confocal microscopy system. Zeiss LSM 700, Zeiss LSM 800, Olympus FV1000, or comparable confocal microscopy systems with multi-channel imaging (488 nm laser, 514–543 nm laser, 633–640 nm laser), z-stacks, and tiling are recommended.

***Optional:*** Other imaging methods, such as Light Sheet Microscopy, can be used to image whole CNS phenotypes in the larval brain in *Drosophila* (Lemon et al., 2015), and could be adapted to this GBM model.

37. Imaging for membrane localized CD8-GFP can clearly visualize glial cell morphology and overall brain morphology (Figure 6).

**Figure 6:**
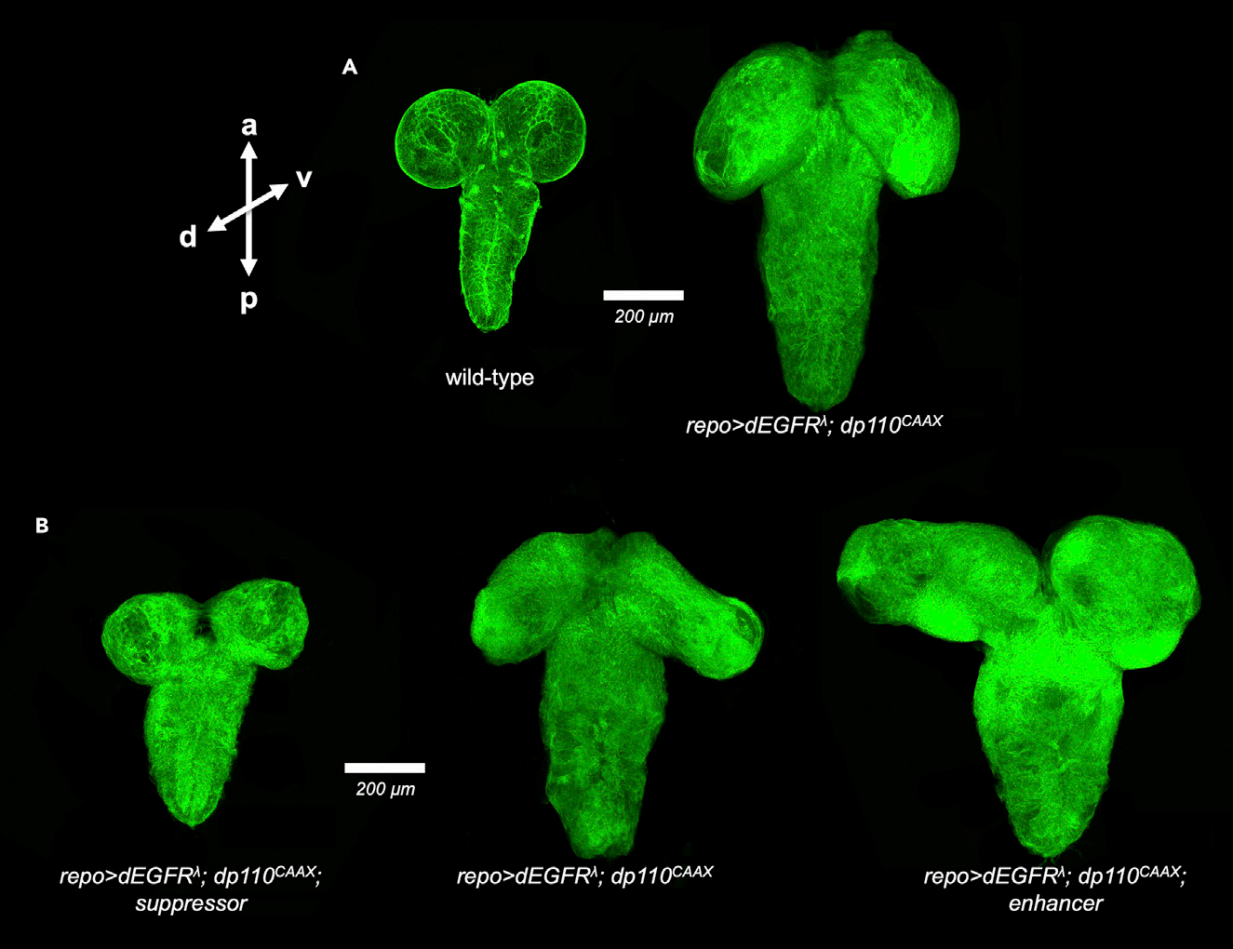
Whole brains from age-matched 3^rd^ instar wandering larvae with EGFR-PI3K GBM phenotypes Optical projections of whole brain-nerve cord complexes from 3rd instar larvae approximately 5 days old, imaged with a 10× objective on a Zeiss 700 confocal microscope. Dorsal view facing forward; anterior up (axes indicated). CD8-GFP (green) expressed by *repo-Gal4* labels glial cell bodies. Scale bar indicates 200 μm. (A) Genotypes: (A) *repo-Gal4 UAS-CD8-GFP* and *UAS-dEGFR*^*λ*^*UAS-dp110*^*CAAX*^*; repo-Gal4 UAS-CD8-GFP.* (B) *UAS-dEGFR*^*λ*^*UAS-dp110*^*CAAX*^*; repo-Gal4 UAS-CD8-GFP/UAS-dRIOK2*^*dsRNA*^*, UAS-dEGFR*^*λ*^*UAS-dp110*^*CAAX*^*; repo-Gal4 UAS-CD8-GFP, and UAS-dEGFR*^*λ*^*UAS-dp110*^*CAAX*^*; repo-Gal4 UAS-CD8-GFP/UAS-Spz*^*act*^*.*
38. Use low magnification imaging at 10× or 25× to visualize the entire brain with z-stacks, which can be used for quantitative analyses of brain size and morphology and for qualitative representative images and optical projections for publications.

**CRITICAL:** Representative low magnification images for experimental tester brain and control brains should be from age-matched samples from the same experiments to control for variability in larval brain growth between batches.

39. Use high magnification imaging at 40× or 63× for imaging entire brain hemispheres and subsections of brain hemispheres to examine desired features, such as glial cell nuclei or cell morphology, preferably using thin optical sections (∼ 1 μm) and an objective with a long working distance to obtain crisp, in focus sections and deep z-stacks.

***Note:*** For imaging larval brain hemispheres, we use a water immersion 40× objective and take z-stacks of 25–45 1 μm slices starting from very cellular superficial areas going into deeper regions that encompass central brain neuropil.

40. For high magnification imaging of *Drosophila* larval brains with glial neoplasia, multiple images will be needed to capture an entire brain hemisphere because brain hemispheres in the GBM model may be larger than the field of view of the objective.

***Note:*** Gain, laser power, and z-stack settings should remain constant while imaging multiple fields from the same brain so that a composite whole brain hemisphere can be stitched together using automated tiling software or manual photo-editing software, such as Photoshop.

**Figure 7:**
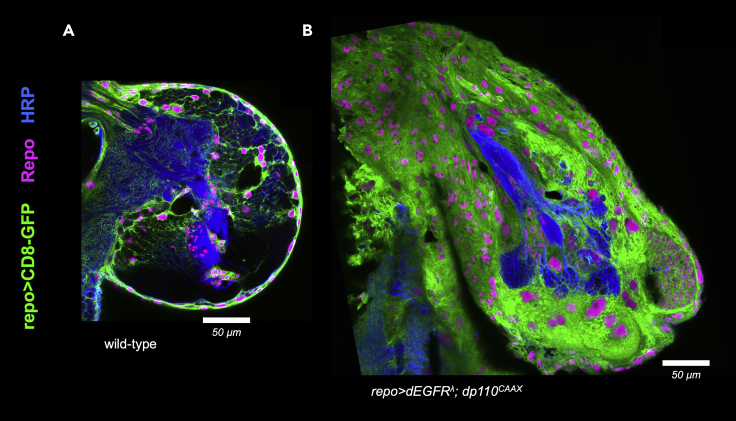
Representative larval brains hemispheres show glial neoplasia present in the EGFR-PI3K GBM model 3 mm optical projections of brain hemispheres, aged-matched 3rd instar larvae, imaged with a 40× objective. Frontal sections, midway through brains. Anterior up; midline left. Repo (magenta) labels glial cell nuclei; CD8-GFP (green) expressed by *repo-Gal4* labels glial cell bodies; anti-HRP (blue) counterstains neurons and neuropil. Scale bar indicates 50 μm. (A and B) Genotypes: (A) *repo-Gal4 UAS-CD8-GFP* and (B) *UAS-dEGFR*^*λ*^*UAS-dp110*^*CAAX*^*; repo-Gal4 UAS-CD8-GFP.*

***Note:*** Representative images for experimental tester samples and age-matched control samples should be matched for brain orientation, section plane, section thickness, and brain region to allow for valid comparisons (Read et al., 2009).

41. For processing high magnification z-stack imaging data, serial section subsets, can be used to create thicker representative optical projections to highlight features of interest, such as glial cell morphology or mitotic figures, to perform quantitative image analyses, and to include as qualitative representative images for publications (Figures 7A and 7B).

***Optional:*** To create representative images of brain hemispheres for publication, we typically select 2–4 serial 1 μm optical section subsets from z-stacks of 40× images and create optical projections from these subsets (Read et al., 2009).

## Expected outcomes

In our *Drosophila* GBM model (*repo> dEGFR*^*λ*^; *dp110*^*CAAX*^, glial-specific overexpression of constitutively active *dEGFR*^*λ*^ and *dp110*^*CAAX*^ induces neoplastic glial cell proliferation that leads to enlargement of the central nervous system (CNS), as compared to wild-type brains (Figure 6A), and late larvae/early pupal lethality. Using genetic crosses, this model can be used to test mutations or UAS-transgene constructs of other genes for their ability ‘enhance’ (worsen) or ‘suppress’ (reduce) glial neoplasia or rescue larval/pupal lethality (Figure 6B), and thereby identify new genes that act in concert with the EGFR and PI3K pathways in tumorigenesis (Chen et al., 2019; Read et al., 2013). UAS-transgenes can be used to overexpress or knockdown candidate genes with RNA-inhibition or clonal mutations (Read et al., 2009), and then examine their effects on the larval brain tumor phenotype (Figure 6B). Using image analysis, the phenotypic impact of any genetic alterations can be qualitatively and quantitatively assessed through evaluating changes in glial cell number by counting the number of anti-Repo-stained glial cell nuclei and through evaluating changes in brain volume and brain/glial cell morphology, as visualized by CD8-GFP expression in glial cell bodies, specific markers or proteins of interest, and/or in anti-HRP staining of neuron cell bodies and neuronal fiber tracts (neuropil) (Figures 7A and 7B). Following such analyses, conclusions can be drawn regarding the impact of the selected genetic interventions on the pathogenic GBM phenotype, and follow-up studies can be performed to investigate the mechanisms by which these interventions exert their effects in *Drosophila* glial neoplasia or in other orthologous GBM model systems. Moreover, this protocol can also be adapted to investigate the influence of additional variables, such as the effects of drugs or nutrients delivered via fly food (Chen and Read, 2019), on growth and development of neoplastic glia.

## Quantification and statistical analysis

1. Quantifying brain volume from low magnification images.
  To gather and analyze quantitative brain size data from low magnification (e.g., 10× images) whole larval brain images, measure the volume of the brain hemispheres using Imaris imaging analysis software (Oxford Instruments).
  a. Import raw z-stack images (.czi files from Zeiss confocal systems) produced by Zeiss LSM systems in ZEN imaging software into Imaris software and convert into Imaris files.
    ***Optional:*** Other software platforms can be used to quantify 3-dimensional larval brain volumes, including other commercial platforms such as Volocity (PerkinElmer), or open source platforms such as Fiji ImageJ (Schindelin et al., 2012), which has a 3D viewer for rendering confocal data that can be used in combination with the freehand draw tool or with the TrakEM2 or Gebiss plugins for volumetric analyses, according to published data analysis pipelines (Kriston-Vizi et al., 2011; Omoto et al., 2015).
  b. In Imaris, select brains, omitting the ventral nerve cord, or other areas of interest to include in total volume calculations from the z-stacks. To do this, select desired brain area using the draw tool every 2–3 slices within each z-stack.
  c. Imaris will calculate only the 3D area (in mm^3^) within the selected regions and give a volumetric value for each selected brain within a z-stack.
  d. To perform statistical analysis of raw volumetric imaging data produced by Imaris, calculate the volumes of 10–20 brains from each experimental tester and matched control group using Imaris, then normalize data for each individual experimental tester brain and each individual control brain to the average for the control group.
    ***Note:*** When analyzing and comparing multiple groups of brains dissected on different days/times, use matched controls for each experimental tester group because larval brain size can vary depending on culture conditions and according to the specific timing of dissections, and age-matched and stage-matched controls are needed to normalize samples.
    ***Optional:*** Separate brains by other categories, such as sex or specific developmental timing, for further subgrouping for statistical analyses.
  e. Analyze normalized data with a t-test for simple pairwise comparisons or with a two-way ANOVA for multiple comparisons. An example grouped normalized data set is shown (Figure 8).

**Figure 8:**
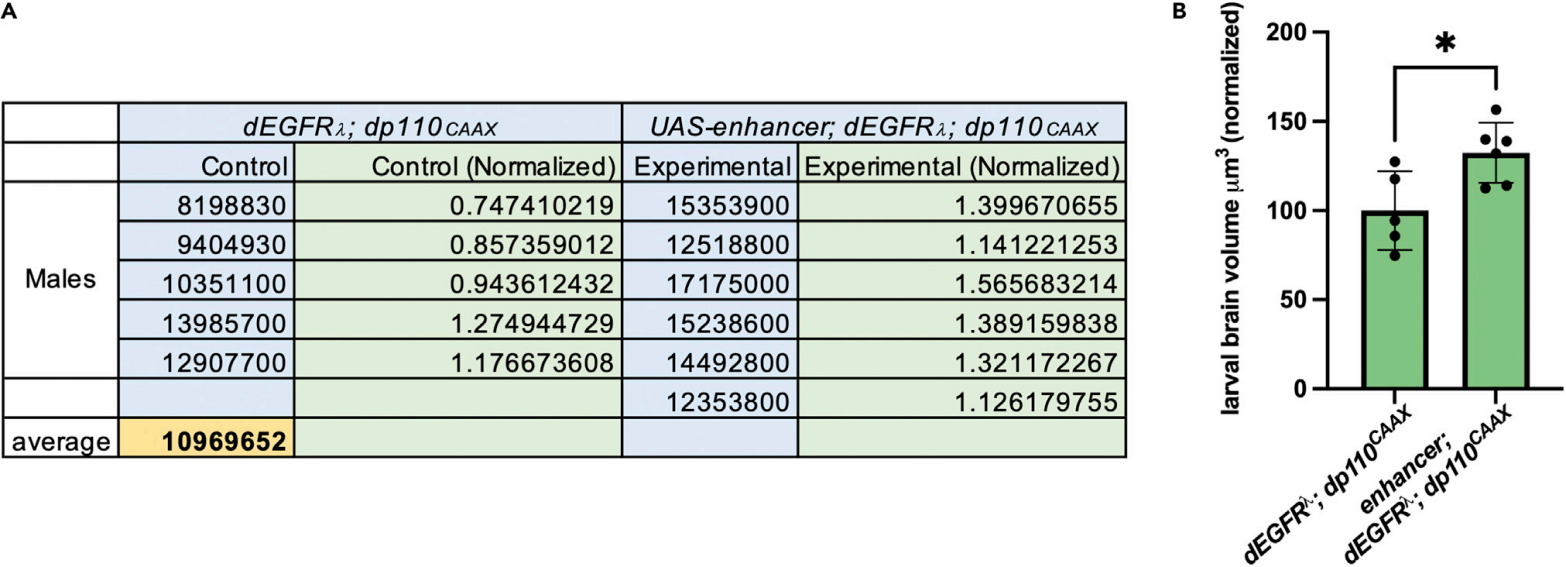
Larval brain volume statistical analysis (A and B) Total volumes (mm^3^) of age-matched 3rd instar larval brains measured using confocal microscopy and Imaris, normalized to GBM controls, and (B) shown in a bar graph; unpaired t-test, ∗p<0.05.
2. Quantifying glial cell number from high magnification images.
  a. To quantify and compare the number of glial cells in each experimental tester sample and control sample, measure the absolute number of glial cells (Repo-positive) in representative high magnification images (e.g., 40× images) matched for brain region and section plane, generated as described above.
  b. Compare experimental samples only to age-matched control samples, and process images for both control and experimental and control images in the same manor (e.g., create projections to represent the same thickness).
    ***Note:*** For counting glial cell numbers, we typically create optical projects select several representative 3 mm thick from 5 or more age-matched and stage-matched experimental tester and control brains and count numbers of glia within each representative optical projection. For an example see (Chen et al., 2019).
  c. To better differentiate glial cell nuclei from other anatomical structures, only visualize the fluorescent channel containing the Repo stain. Manually count the number of glial cells using Zen, Image J, or Photoshop.
    ***Note:*** Due to how dense and irregularly shaped the glial cell nuclei are in this *Drosophila* GBM model, automated cell counting is not appropriate or feasible.
  d. Results are reported as total number of glial cells per brain hemisphere per optical projection.
  e. Statistical and graphical analyses should be performed on data from at least 5 animals per genotype to reach statistical significance. T-tests are used for simple pairwise comparisons, and two-way ANOVA are used for multiple comparisons.

## Limitations

This protocol allows researchers to characterizeneoplastic *Drosophila* larval brain growth and development in a snapshot of time. Therefore, age matching procedures need to be employed such that experimental tester animals are always compared to synchronous age-matched control animals. Culture conditions including food consistency and formulation, humidity, temperature, and animal numbers all affect both the overall larval growth rate and the GBM phenotype. Moreover, higher temperatures enhance Gal4 transcriptional activity and therefore cause a more severe tumor phenotype in larvae. Additionally, both control and experimental tester larval progeny should be dissected synchronously on the same day so as not to give one group a growth advantage that may bias neoplastic phenotypes towards one genotype and thereby invalidate results. Conditions used for fixation, immunohistochemical staining, and confocal imaging affect outcomes. Blinded labeling of experimental tester and control samples is recommended to correct for any bias in sample processing, image analysis, and/or statistical analyses.

## Troubleshooting

### Problem 1

Poor recovery of 3^rd^ instar larval progeny with neoplastic brains from among F1 cross progeny.

### Potential solution

Low numbers of F1 progeny with neoplastic brains generally occur in three circumstances: 1) F1 larvae are either overly crowded or are not age-matched or stage-matched, 2) experimental F1 larvae may die early due to the lethal effect of an additional mutation or UAS- transgene on glia, or 3) F1 larvae may die or not mature due to problems with culture conditions and/or formulation of larval food.

To prevent overcrowding, ensure that embryo clutches are collected every 12–24 h by flipping parental crosses into fresh vials every 12–24 h. This will allow for accurate age matching of growth among larvae from the same crosses and between larvae from experimental and control crosses. Ensure sufficient mated females are added per vial (∼25 or more) at the outset, and add fresh females to maintain ample parental crosses for all genotypes. If F1 progeny are collected over several days in the same vial, GBM model larvae typically show an exacerbated stress response to overcrowding, and wander prematurely, likely due to the involvement of the Insulin signaling pathway in glial development and neoplastic glia transformation (Read, 2018). Additionally, to improve embryo numbers, a sprinkle of dry yeast may be added to enhance parental flies’ egg laying. To determine if experimental animals show early lethality, embryos can be hatched and 1^st^ and 2^nd^ instar GBM larvae can be examined for phenotypes and lethal phase, and CD8-GFP fluorescence can be used to monitor phenotypes in live animals as they mature. Finally, problems with larval recovery are often related to culture conditions such that temperature, humidity, and food quality must be evaluated. For example, if fly food is too wet, larvae may die and/or not grow; a small strip of filter paper in the vial will absorb excess water. Alternately, GBM model larvae can be more sensitive to overly dry or hard food; effects of oncogenes on glial function can compromise locomotion such that GBM larvae may struggle to work their food and become more vulnerable to stress. Because Gal4 is temperature sensitive, low temperatures can suppress the GBM phenotype.

### Problem 2

The brain tissue does not fix properly (step 7).

### Potential solution

Fixation of the larval brain tissue is sensitive to pH and osmolarity; therefore, ensure all dilution calculations are correct when preparing a solution of 4% paraformaldehyde in 1× PBS. When fixing large numbers of brains, ensure all brains are submerged in fixative and that they do not float to the top or stick to the sides of the well, as this will prevent paraformaldehyde from evenly penetrating tissues. If tissues are not fixing well, increase the amount of fixative per well and/or decrease the number of brains per well. Furthermore, thorough washing of brains after fixation and between antibody staining steps is important for optimal labeling. Tissue that did not fix well can be identified during mounting and confocal imaging by the presence of ragged and shriveled brain hemispheres and tissue degradation.

### Problem 3

Immunofluorescence staining or imaging results in brain tissue are suboptimal (steps 15–22).

### Potential solution

Firstly, ensure that the combination of primary and secondary antibodies used have minimal or no spectral overlap. To control for this, run control immunofluorescence stains using each antibody separately, and compare the results to your multiplex image. If there is overlap, adjust filters and other confocal settings to minimize overlap.

More commonly, high background and/or low intensity immunofluorescence can make imaging and imaging analysis difficult. This is because the performance and specificity of primary antibodies can vary. To reduce background, primary antibodies can be pre-absorbed against fixed larval carcasses, which can deplete ‘sticky’ non-specific contaminants.

To limit faint or dim staining, before adding the primary antibody ensure maximum removal of 1× PBS .3% Triton wash solution so as not to further dilute the antibody concentration. Primary antibodies can also show poor penetration into the brain tissues, yielding strong staining in more superficial areas of the brain but faint/low staining of deeper interior areas. To improve penetration, incubate brains in primary antibody for a minimum of 48 h. Increasing the antibody incubation time beyond 72 h typically does not improve staining quality but may instead create higher background in samples. Staining procedures, such as antibody dilution, can be further optimized through standard troubleshooting.

If primary antibody staining procedures have been optimized, imaging and image processing procedures can then be optimized. To reduce imaging background, the pinhole in the confocal system can be reduced to limit non-specific signal from the antibody stain. For more diffuse and dynamic staining, optical projections of z-stack images several microns thick can intensify the specific signal and provide more anatomical context to the staining pattern, and may thus yield informative images. For particulate background, particularly in nuclei, optical projections of 2–3 microns can be filtered or de-speckled using image processing software, although care must be taken to not distort primary imaging results.

## Resource availability

### Lead contact

Further information and requests for resources and reagents should be directed to and will be fulfilled by the lead contact, Renee D. Read (renee.read@emory.edu).

### Materials availability

This study did not generate new unique reagents. All *Drosophila* stocks required to produce larval glioblastoma models can be requested from the Bloomington *Drosophila* Stock Center (https://bdsc.indiana.edu/index.html) and from originating investigators (*UAS-dEGFR*^*λ*^ on the X chromosome is available from Dr. Gertrud Schupbach (schupbac@princeton.edu)). Identifiers for all *Drosophila* stocks are provided in the key resources table.

## Data Availability

This study did not generate/analyze any unique code. Original data for procedures developed in this paper are available in Read et al., (2009). [https://doi.org/10.1371/journal.pgen.1000374]. Zeiss ZEN Black, Imaris Image Analysis software, and GraphPad Prism are commercially available.

## References

[bib1] Brennan C.W., Verhaak R.G.W., McKenna A., Campos B., Noushmehr H., Salama S.R., Zheng S., Chakravarty D., Sanborn J.Z., Berman S.H. (2013). The somatic genomic landscape of glioblastoma. Cell.

[bib2] Chen A.S., Read R.D., Deng W. (2019). The Drosophila Model in Cancer.

[bib3] Chen A.S., Wardwell-Ozgo J., Shah N.N., Wright D., Appin C.L., Vigneswaran K., Brat D.J., Kornblum H.I., Read R.D. (2019). Drak/STK17A drives neoplastic glial proliferation through modulation of MRLC signaling. Cancer Res..

[bib4] Karabasheva D., Smyth J.T. (2020). Preparation of Drosophila larval and pupal testes for analysis of cell division in live, intact tissue. J. Vis. Exp..

[bib5] Kriston-Vizi J., Thong N.W., Poh C.L., Yee K.C., Ling J.S.P., Kraut R., Wasser M. (2011). Gebiss: an ImageJ plugin for the specification of ground truth and the performance evaluation of 3D segmentation algorithms. BMC Bioinf..

[bib6] Lemon W.C., Pulver S.R., Höckendorf B., McDole K., Branson K., Freeman J., Keller P.J. (2015). Whole-central nervous system functional imaging in larval Drosophila. Nat. Commun..

[bib7] Omoto J.J., Yogi P., Hartenstein V. (2015). Origin and development of neuropil glia of the Drosophila larval and adult brain: two distinct glial populations derived from separate progenitors. Dev. Biol..

[bib8] Read R.D. (2018). Pvr receptor tyrosine kinase signaling promotes post-embryonic morphogenesis, and survival of glia and neural progenitor cells in Drosophila. Development.

[bib9] Read R.D., Cavenee W.K., Furnari F.B., Thomas J.B. (2009). A drosophila model for EGFR-Ras and PI3K-dependent human glioma. PLoS Genet..

[bib10] Read R.D., Fenton T.R., Gomez G.G., Wykosky J., Vandenberg S.R., Babic I., Iwanami A., Yang H., Cavenee W.K., Mischel P.S. (2013). A kinome-wide RNAi screen in Drosophila Glia reveals that the RIO kinases mediate cell proliferation and survival through TORC2-Akt signaling in glioblastoma. PLoS Genet..

[bib11] Schindelin J., Arganda-Carreras I., Frise E., Kaynig V., Longair M., Pietzsch T., Preibisch S., Rueden C., Saalfeld S., Schmid B. (2012). Fiji: an open-source platform for biological-image analysis. Nat. Methods.

